# Disordered Glass Nanowire Substrates Produce in Vivo‐Like Astrocyte Morphology Revealed by Low‐Coherence Holotomography

**DOI:** 10.1002/advs.202513424

**Published:** 2025-11-03

**Authors:** Pooja Anantha, Anoushka Gupta, Joo Ho Kim, Emanuela Saracino, Piyush Raj, Ivano Lucarini, Swati Tanwar, Jessica Chen, Luo Gu, Jay Agrawal, Annalisa Convertino, Ishan Barman

**Affiliations:** ^1^ Department of Mechanical Engineering Johns Hopkins University Baltimore MD 21218 USA; ^2^ Department of Materials Science and Engineering Johns Hopkins University Baltimore MD 21218 USA; ^3^ Institute for NanoBioTechnology Johns Hopkins University Baltimore MD 21218 USA; ^4^ Institute for Organic Synthesis and Photoreactivity (ISOF) National Research Council Via P. Gobetti 101 Bologna I‐40129 Italy; ^5^ Institute for Microelectronics and Microsystems National Research Council via Fosso del Cavaliere 100 Rome 00133 Italy; ^6^ Department of Biology Johns Hopkins University Baltimore MD 21218 USA; ^7^ Translational Tissue Engineering Center Johns Hopkins University School of Medicine 400 N. Broadway Baltimore MD 21231 USA; ^8^ Department of Radiology VA Hudson Valley Health Care System Wappingers Falls NY 12590 USA; ^9^ Department of Oncology Johns Hopkins University Baltimore MD 21287 USA; ^10^ The Russell H. Morgan Department of Radiology and Radiological Science Division of Cancer Imaging Research Johns Hopkins University School of Medicine Baltimore MD 21205 USA

**Keywords:** astrocytes, cell–substrate interactions, glass nanowire substrates, label‐free imaging, low‐coherence holotomography

## Abstract

Astrocytes are essential for preserving homeostasis of the central nervous system (CNS). They regulate synaptic activity and interact with the extracellular milieu via their distinctive, star‐like morphology. However, there is a lack of detailed understanding of astrocyte morphology, particularly of the in vivo phenotype that is difficult to replicate in vitro and quantify using conventional imaging techniques without exogenous labels. This study marks the first demonstration of low‐coherence holotomography (LC‐HT), a label‐free imaging technique, for 3D quantitative assessment of astrocyte morphology cultured on nanostructured substrates, which typically presents challenges for phase‐based imaging. Crucially, it is shown that disordered glass nanowire (NW) substrates can induce in vivo‐like astrocyte morphology in cultured rat cortical astrocytes. Compared to traditional glass substrates, astrocytes grown on disordered glass NWs substrates exhibit enhanced process branching and greater total arbor length – features typically observed in their natural, in vivo state of advanced maturation. By combining disordered glass NW substrates with LC‐HT, the approach uniquely enables high‐fidelity, label‐free visualization of complex astrocytic morphologies, providing a powerful platform to study how nanoscale environmental cues shape astrocyte development.

## Graphical abstract

Low‐coherence holotomography reveals that disordered nanowire substrates guide astrocyte morphology toward branched, in vivo–like star‐shaped morphology, emphasizing the role of biophysical cues in shaping neural cell behavior. The developed nanostructured platform offers a new route to study astrocytes for neurobiology studies.

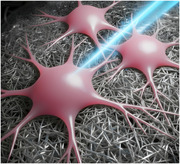

## 1 Introduction

Growth, development, and homeostasis in multicellular organisms rely on the precise control of cellular form. Cell shape is not merely a passive consequence of internal structure; it actively reflects cellular function, state, and its interaction with the surrounding microenvironment. Changes in cell morphology arise from both internal programs and external cues,^[^ ^1^, ^2^ ^]^ which make morphology a sensitive indicator of development, maturation, and disease. Mechanosensitive adhesion complexes mediate these responses, allowing cells to sense and respond to physical features in their environment across spatial and temporal scales.^[^ ^2^, ^3^, ^4^, ^5^ ^]^ Understanding how cells adopt specific morphologies in response to their surroundings is central to decoding complex biological processes and their dysregulation in disease.

Nanostructured materials offer a powerful way to control the physical microenvironment at the nanoscale. In particular, nanopillars and nanowires (NWs) provide topographical and mechanical cues that can reorganize the cytoskeleton, limit spreading, and mimic extracellular matrix (ECM) fibrillar structure. These cues frequently promote the extension of long, thin processes in neurons,^[^ ^6^, ^7^, ^8^, ^9^, ^10^, ^11^ ^]^ resembling their in vivo morphologies. This capability is especially important for in vitro models of the central nervous system (CNS) where cell shape is tightly coupled to their function and dysfunction.^[^ ^12^, ^13^, ^14^ ^]^ NW arrays also serve as high‐density electrode platforms, and enable neural activity recording with exceptional spatial and temporal resolution,^[^ ^9^, ^10^, ^11^, ^15^, ^16^, ^17^, ^18^ ^]^ thus merging structural and functional insights in vitro.

While neurons have long been the focus of neurotechnology and in vitro modeling, there is growing recognition that non‐neuronal glial cells, especially astrocytes, play equally critical roles in CNS function. Astrocytes regulate ion balance, maintain the blood–brain barrier, modulate synaptic transmission, and provide metabolic and structural support to neurons. These diverse functions are intimately linked to their stellate morphology. In vivo, astrocytes display a highly complex and specialized structure, with fine, branched processes extending in three dimensions to define individual, non‐overlapping anatomical domains. Within these domains, each astrocyte establishes thousands of contacts with synapses, blood vessels, and neighboring glial cells, thereby creating a tightly regulated microenvironment. Through these extensive interactions, astrocytes act as active regulators of neuronal circuits rather than passive supporting cells.^[^ ^19^ ^]^ Changes in astrocyte morphology often accompany development, injury, or disease.^[^ ^10^, ^20^ ^]^

Replicating astrocytic morphology in vitro is therefore an indispensable first step toward more physiologically relevant brain models: while morphology alone cannot capture the full spectrum of astrocyte functionality, it provides the structural foundation upon which their diverse biochemical and electrophysiological roles depend. However, conventional 2D culture systems fail to preserve such morphology,^[^ ^21^ ^]^ leading to astrocytes adopting a simplified, flattened appearance that obscures their physiological roles. In this context, nanostructured substrates such as NW mats represent a promising strategy for restoring in vivo‐like morphological complexity^[^ ^22^ ^]^ and enabling systematic investigations of structure–function relationships in astrocytes.

However, to fully understand how nanoscale cues influence astrocyte morphology and maturation, we need imaging tools that can capture subtle, 3D structural changes with high precision and without perturbing the cell. Optical diffraction tomography (ODT), in principle, addresses this need by providing label‐free, high‐resolution 3D reconstructions of a cell's refractive index (RI) distribution – effectively offering quantitative maps of cell morphology, volume, and dry mass in real time.^[^ ^23^, ^24^, ^25^, ^26^, ^27^, ^28^, ^29^, ^30^ ^]^ Unlike traditional fluorescence microscopy, ODT operates on unlabeled specimens, avoiding issues such as phototoxicity, photobleaching, or label‐induced artifacts. Its ability to reveal fine structural features and dynamic changes without contrast agents makes it ideally suited for analyzing morphologically complex cells, such as astrocytes, in a physiologically relevant context. Our recent work has leveraged ODT to dynamically monitor primary rat cortical astrocytes in vitro on flat glass substrates over several days, enabling quantitative assessment of morphology at high spatial resolution.^[^ ^23^ ^]^

We observed that astrocytes cultured on standard smooth glass substrates adopt an unnaturally rounded shape, lacking the branched, stellate morphology seen in vivo. This motivates the use of disordered NW scaffolds thatintroduce biomimetic topographical cues known to promote astrocyte maturation. A critical challenge, however, lies in imaging astrocytes cultured on nanostructured materials: most of them are opaque and highly scattering, precluding the transmission‐based imaging techniques like ODT. Furthermore, traditional ODT systems – reliant on coherent laser illumination – require background calibration over smooth, homogeneous regions to reconstruct phase maps accurately. The inherent randomness of NW substrates disrupts this calibration: scattered light from the irregular surface generates unpredictable background signals that cannot be reliably subtracted, resulting in reconstruction artifacts and severe phase unwrapping errors.

**Figure 1:**
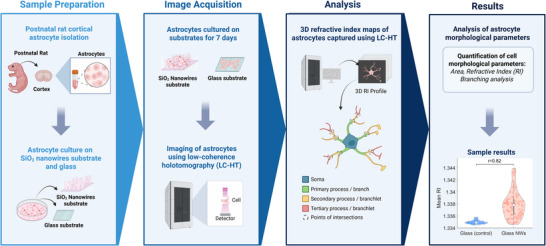
*Schematic overview of the experimental workflow*. Primary rat cortical astrocytes were cultured on fully transparent NW substrates and conventional flat glass substrates as controls.LC‐HT was used to acquire high‐resolution 3D RI maps of the cells. Quantitative morphological analyses were then performed on the RI maps to assess structural differences among astrocyte populations grown on different substrates.

Here, we present a novel imaging platform that combines low‐coherence holotomography (LC‐HT) with fully transparent disordered glass NW substrates to enable high‐fidelity, label‐free visualization of astrocyte morphology in vitro (**Figure** **1**). In contrast to traditional ODT systems, LC‐HT uses incoherent illumination sources like LEDs and a self‐interference‐based phase retrieval scheme, eliminating the need for background calibration and dramatically reducing speckle noise.^[^ ^31^, ^32^, ^33^ ^]^ This allows robust 3D reconstruction of cells even in the presence of complex, irregular substrates. By leveraging transparent NW scaffolds, we maintain compatibility with transmission‐based imaging while providing the nanoscale topographical cues that promote astrocyte maturation. When cultured on these biomimetic substrates, astrocytes exhibit pronounced branching and arborization, hallmarks of their in vivo morphology – that are absent on flat glass. This platform overcomes longstanding barriers to imaging on nanostructured materials and enables, for the first time, quantitative analysis of in vivo‐like astrocyte morphology in a controlled in vitro setting. By combining transparent NWs with LC‐HT, we provide a physiologically relevant and quantitative framework that represents a critical step toward constructing advanced in vitro models of astrocyte function.

## 2 Results and Discussion

### 2.1 Engineering Transparent NW Substrates for LC‐HT

To enable label‐free, high‐resolution imaging of astrocyte morphology using LC‐HT, it is essential to develop a substrate that offers both biomimetic nanoscale topography and optical transparency. Traditional NW platforms often obstruct light transmission due to their opacity, making them incompatible with interferometric imaging methods. Here, we report the fabrication and characterization of fully transparent disordered glass NW substrates that overcome these limitations.

**Figure 2:**
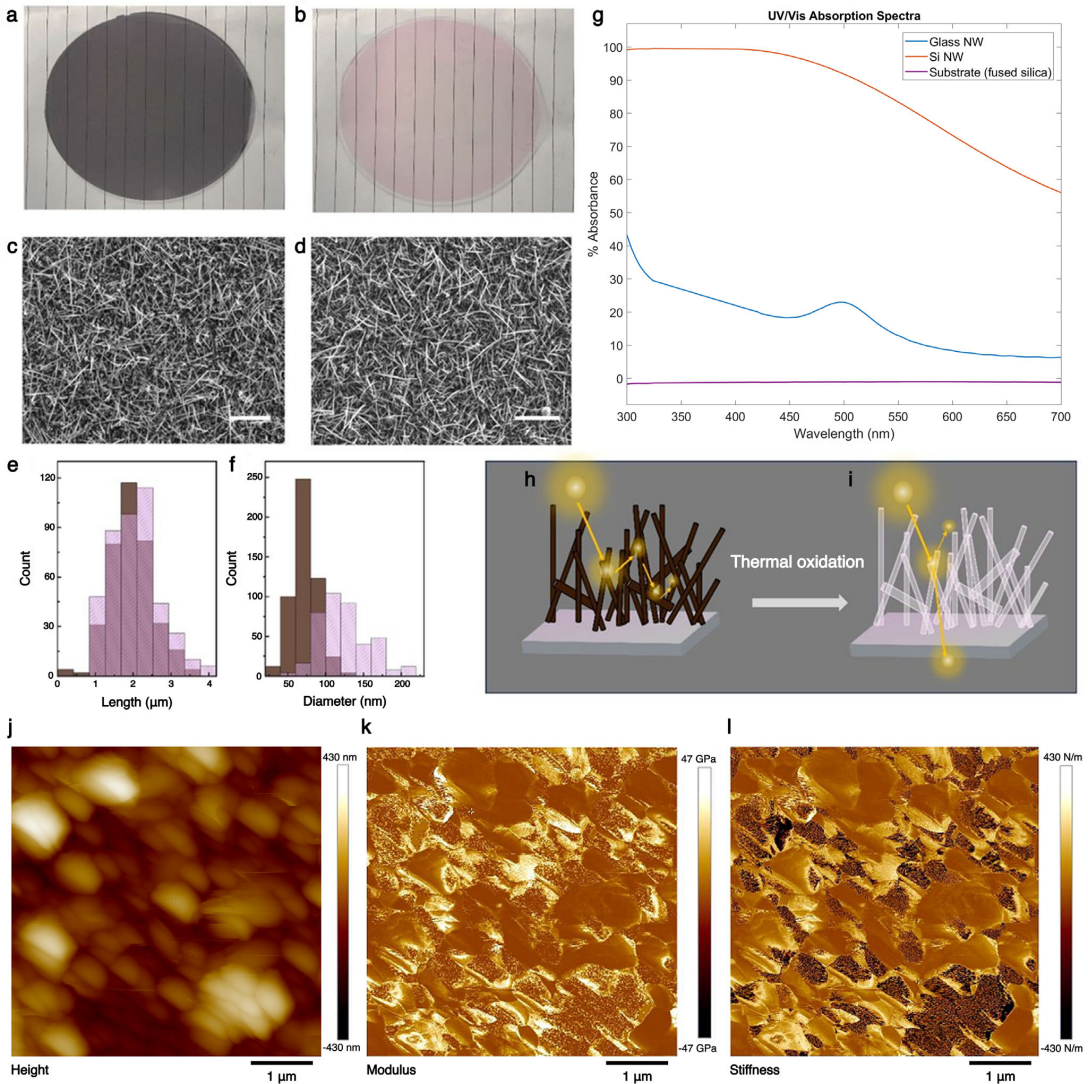
*Characterization of Si NW and glass NW substrates*. a,b) Photographs of the NW substrates before (a) and after (b) thermal oxidation, showing the transition from opaque Si NWs to transparent glass NWs. c,d) SEM images of as‐grown (c) and thermally oxidized Si (glass) NWs (d). Scale bar: 5 µm. e,f) Histograms showing length (e) and diameter (f) distributions of Si NWs (brown) and glass NWs (pink). g) Absorbance spectra, A(λ), of as‐grown Si NWs (orange), glass NWs (blue), and bare fused silica substrate (purple), measured from 300–700 nm. h,i) Schematic illustrations of light propagation through the NW mats: h) Si NWs showing strong multiple scattering and light trapping; i) glass NWs acting as a quasi‐transparent medium j–l) AFM measurements showing height, Young's modulus, and stiffness distribution for a representative region of the glass NW substrate.

Transparent nanostructured substrates composed of SiO_2_ NWs (referred to as glass NWs) were fabricated via thermal oxidation of silicon NWs (Si NWs). Initially, Si NWs were grown on fused silica substrates using plasma‐enhanced chemical vapor deposition (PECVD), producing opaque, brown‐colored samples (**Figure** **2**a). These NWs were then thermally oxidized at 980 °C in an oxygen‐rich atmosphere overnight, converting the silicon to amorphous SiO_2_.^[^ ^34^, ^35^, ^36^ ^]^ The resulting substrates appeared transparent with a faint reddish hue, as shown in Figure 2b. Scanning electron microscopy (SEM) images before and after oxidation (Figure 2c,d) revealed dense, disordered mats of randomly oriented NWs with tapered geometries. Quantitative comparisons of length and diameter distributions (Figure 2e,f) showed similar NW lengths (1–3 µm), but the glass NWs exhibited increased diameters (80–180 nm) relative to the original Si NWs (50–80 nm), consistent with volumetric expansion during oxidation due to the molar volume difference between Si and SiO_2_.^[^ ^37^ ^]^ Importantly, this transformation did not compromise the structural integrity of the NWs.

To evaluate optical properties, we measured transmittance (T) and reflectance (R) across the spectral range of 300–700 nm, and computed absorbance as A(λ) = 100 – T(λ) – R(λ). As shown in Figure 2g, in air, the Si NW mats (orange line) exhibited strong absorbance, whereas glass NW mats (blue line) showed significantly lower absorbance (≈20% across the visible range) with a broad peak near 500 nm, imparting the reddish appearance observed in Figure 2b. The bare fused silica substrate (purple line) displayed minimal absorbance. The high absorbance in Si NWs arises from light trapping, a common phenomenon in semiconductor NW arrays,^[^ ^38^, ^39^ ^]^ where incident light undergoes multiple scattering events (Figure 2h) when the NW diameter *d*_NW_ is approximately *λ/10* (λ being the incident light wavelength). In our samples, the combination of NW dimensions and tapering enhances light scattering, increasing path length and absorption below Si's bandgap (λ < 1.1 µm), thus producing the characteristic dark appearance.

In contrast, glass NWs exhibited good transparency due to the inherent optical properties of SiO_2_, which is transparent down to ≈200 nm. Moreover, considering the typical RI of SiO_2_ (1.45–1.54) in the visible range,^[^ ^40^ ^]^ immersion of the glass NW mats in cell culture medium results in a small RI mismatch with the surrounding environment (Δn ≈0.11–0.21, assuming n ≈1.34 for both culture media and phosphate buffered saline).^[^ ^41^ ^]^ This weak dielectric contrast suppresses Fresnel reflections at the NW–medium interfaces and substantially reduces internal scattering events, thereby enabling incident light to traverse the NW mat with negligible attenuation. Consequently, under culture conditions, the glass NW mat optically approximates a homogeneous, transparent medium (Figure 2i). Additionally, the presence of gold nanoparticles used to catalyze Si NW growth (via the vapor‐liquid‐solid mechanism^[^ ^42^, ^43^, ^44^ ^]^) likely contributes to the absorption peak at 500 nm, corresponding to localized surface plasmon resonance.

Finally, to assess mechanical properties, we conducted atomic force microscopy (AFM) measurements (Figure 2j,i). The AFM maps (Figure 2j–l) confirmed that NWs had significant spatial variation in all the three properties – height, modulus, and stiffness. Specifically, the variation in height confirms that the substrates provide geometrically similar (non‐planar) adhesion sites for cell attachment as found in previous studies.^[^ ^6^, ^7^, ^8^, ^9^, ^10^, ^11^ ^]^

### 2.2 Visualizing and Quantifying Astrocyte Morphogenesis on Glass NW Substrates Using LC‐HT

**Figure 3:**
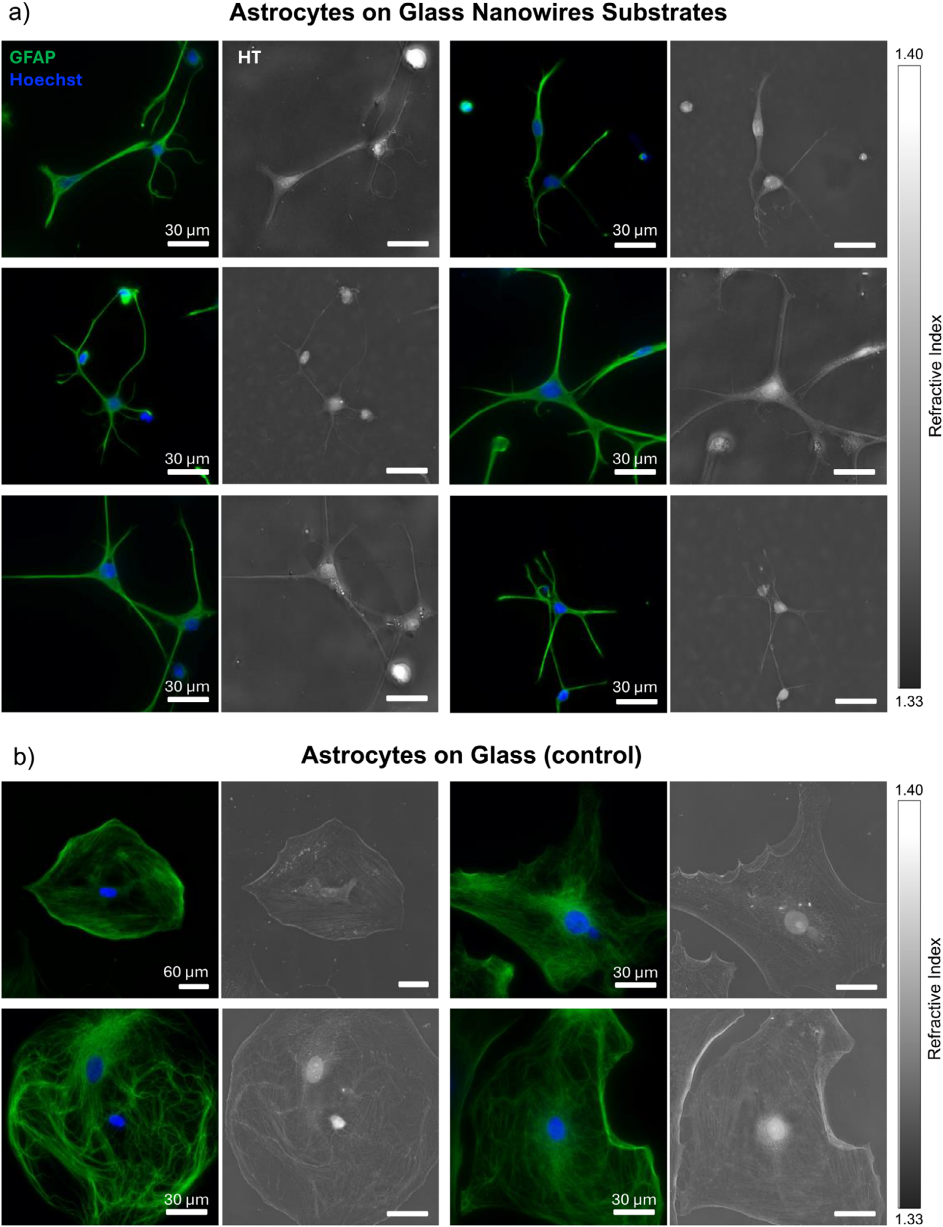
Correlative LC‐HT and fluorescence images of day 7 astrocytes on glass NWs substrates (a) and on glass (b). Fluorescence images show glial fibrillary acidic protein (GFAP) (green) and nucleus (blue) expressions along with the corresponding maximum intensity profile (MIP) images to the right of them. Bars represent the range of RI values.

To investigate how substrate topography influences astrocyte development, we cultured primary rat cortical astrocytes on disordered glass NW substrates and conventional flat glass controls for seven days. Using correlative LC‐HT and fluorescence, we captured high‐resolution 3D RI maps of the cells to assess morphological differences (**Figure** **3**a,b). This platform enabled, for the first time, quantitative imaging of astrocytes on nanostructured surfaces—an achievement made possible by the optical clarity of the latter, and the calibration‐free nature and reduced speckle noise of the LC‐HT system.

Striking differences were observed in astrocyte morphology across the two substrates. On flat glass surfaces, astrocytes adopted a polygonal, flattened morphology with limited extension of processes (Figure 3b) – an outcome commonly observed in conventional 2D culture systems. This constrained geometry arises from the absence of topographical or mechanical cues, leading to restricted cytoskeletal reorganization and impaired polarization. As a result, the astrocytes fail to exhibit their characteristic radial symmetry and instead spread laterally with only short, stubby protrusions. In contrast, astrocytes cultured on glass NWs displayed a dramatically different morphology: a stellate architecture with elongated processes radiating symmetrically from the soma, more akin to their native presentation in vivo (Figure 3a). These star‐like forms are widely recognized as markers of advanced functional maturation, enabling astrocytes to establish broad spatial territories for interaction with neurons, blood vessels, and other glia.

**Figure 4:**
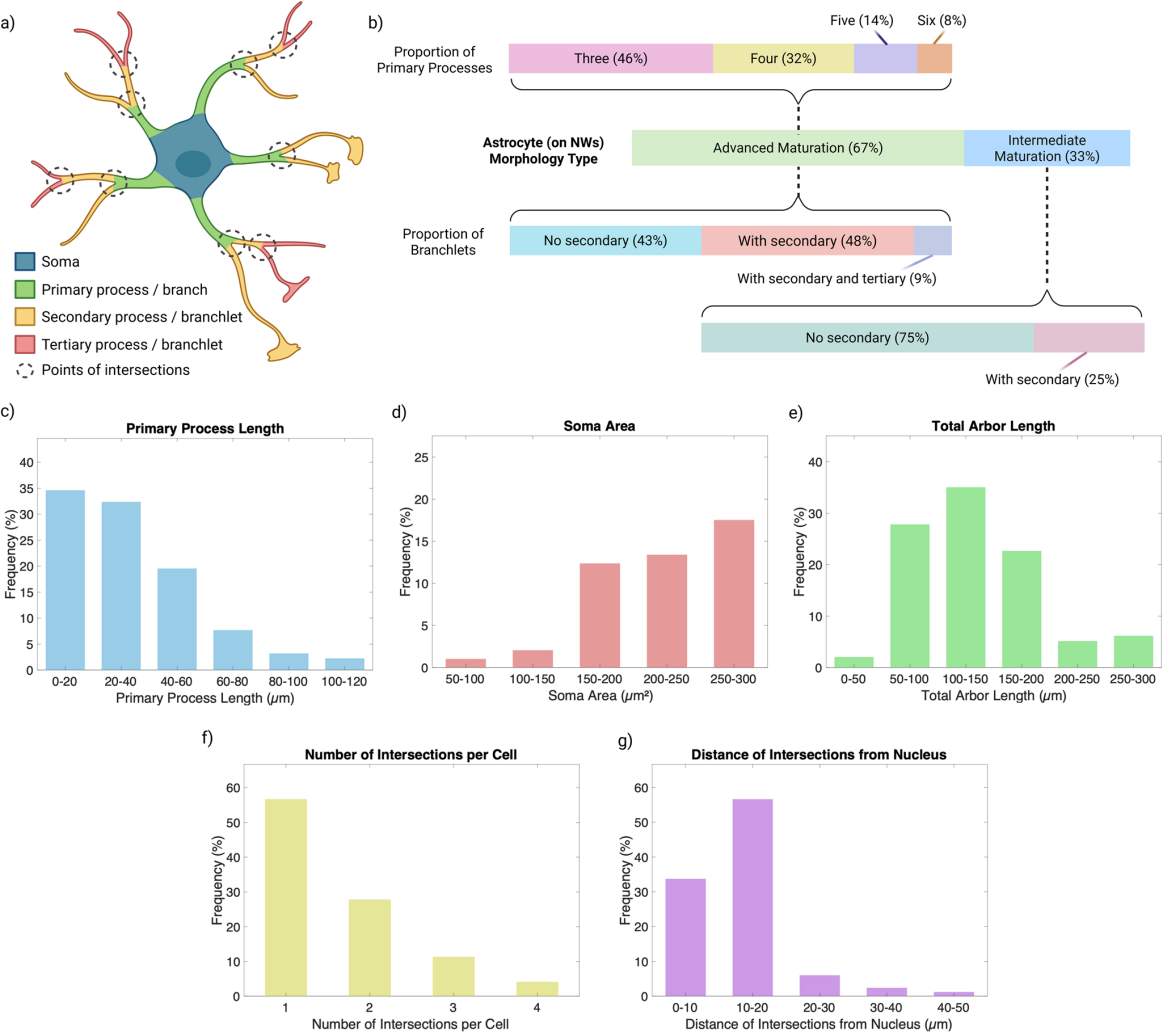
*Quantitative analysis of astrocyte morphogenesis on glass NW substrates*. a) Schematic illustration of key morphological features observed in an advanced maturation astrocyte, including primary processes and higher order branchlets. b) Proportions of astrocytes in intermediate (2 primary processes) versus advanced (3–6 primary processes) maturation states; distributions of primary process count within the advanced group; and relative occurrence of secondary and tertiary branchlets. c–g) Morphometric parameters extracted from LC‐HT images: c) distribution of primary process lengths, d) soma area, e) total arbor length of primary processes, f) number of process intersections per astrocyte, and g) radial distance from each intersection to the nucleus. Sample size: n = 96 cells.

LC‐HT imaging enabled not only visualization but also quantification of astrocytic features including primary processes (those extending directly from the soma) and higher‐order branchlets (secondary and tertiary extensions) (**Figure** **4**a). The number, length, and spatial distribution of these branches are critical morphological readouts that correlate with astrocyte responsiveness, signaling capacity, and interaction with synaptic and vascular targets. Astrocytes exhibiting only two primary processes are often interpreted as being in an intermediate maturation state, whereas those with more complex, highly branched arbors reflect advanced maturation.^[^ ^45^, ^46^ ^]^

Figure 4b–g provides a detailed morphometric analysis of the morphological states observed using LC‐HT. Astrocytes cultured on the glass NW substrates developed up to six distinct primary processes, with over 50% of the population exhibiting at least secondary branchlets, which is close to the >6 primary processes typically observed in vivo.^[^ ^46^ ^]^ This degree of complexity has not been previously reported in vitro on flat glass^[^ ^23^ ^]^ and supports the hypothesis that nanostructured substrates provide key physical cues to guide morphological maturation. The presence of multiple process levels and extended arbor lengths suggests structural maturity and a readiness for functional engagement with the surrounding microenvironment. The ability to visualize these structures in 3D, without staining or perturbation, highlights the unique capabilities of LC‐HT for capturing these morphogenetic events.

Furthermore, LC‐HT allowed us to quantify the number of branch intersections per cell and the distance of each intersection from the nucleus – metrics commonly used to characterize astrocyte territorial coverage and topological complexity.^[^ ^47^, ^48^, ^49^ ^]^ A higher number of intersections and longer distances from the soma are indicative of cells with more expansive and interconnected arbors, which are thought to reflect enhanced capacity for synaptic ensheathment, potassium buffering, and metabolic support. These spatial metrics are particularly important considering astrocytes’ roles in regulating synaptic function, maintaining ion homeostasis, and facilitating neurovascular coupling.^[^ ^45^, ^50^, ^51^ ^]^

To further understand the distribution of astrocyte morphological states on NW substrates, we classified cells based on their number of primary processes. As shown in Figure 4b, ≈33% of astrocytes exhibited only two processes and were classified as being in an intermediate maturation state. In contrast, 67% of the population displayed three to six processes, consistent with advanced maturation. Among this group, the majority (46%) had three primary processes, followed by 32% with four, 14% with five, and 8% with six processes. The number of primary processes serves as a key metric of morphogenetic progression and functional specialization; increased process count typically corresponds to greater territorial coverage and higher levels of synaptic engagement. We also observed that 48% of astrocytes extended secondary branchlets, and 9% even formed tertiary processes, indicating substantial arborization complexity not typically achievable on flat substrates. These findings underscore the role of NW scaffolds in promoting both morphological and potentially functional maturation, positioning them as an effective platform for developing physiologically relevant in vitro CNS models.

Figure 4c–g further details the morphometric parameters extracted from the LC‐HT data. The distribution of primary process lengths (Figure 4c) showed that most extensions fall within the 0–40 µm range, with a peak in the 0–20 µm bin. This observation aligns with previous studies demonstrating that topographical features such as nanoscale ridges or pillars can induce cytoskeletal remodeling, promoting elongation and branching.^[^ ^52^ ^]^ The enhanced mechanotransduction triggered by NW contact can activate integrin‐mediated signaling cascades that regulate actin polymerization and microtubule extension, leading to more elaborate process formation.^[^ ^53^ ^]^ In vivo, elongated processes facilitate astrocyte interactions with synaptic and vascular elements, contributing to metabolic support, neurotransmitter clearance, and blood‐brain barrier regulation.^[^ ^54^ ^]^ Thus, the increased process lengths observed on NW substrates suggest a shift toward phenotypes associated with greater structural and functional integration. The soma area distribution (Figure 4d) was also analyzed to assess cellular compactness and growth. Most astrocytes on NW substrates displayed moderate soma sizes between 200 and 250 µm^2^, with fewer cells at the extremes (< 150 µm^2^ or > 250 µm^2^). This bell‐shaped distribution is consistent with a healthy, non‐hypertrophic cell population and reinforces the notion that NWs support morphologically stable, mature astrocytes.

The total arbor length (Figure 4e) offers a key measure of the spatial extent of astrocyte branching. Astrocytes cultured on NW substrates exhibited arbor lengths predominantly in the 100–200 µm range, with the highest frequency between 100 and 150 µm. These values are consistent with cells in a state of advanced maturation and reflect an increased capacity for spatial integration within a neural environment. Longer and more complex arbors suggest that astrocytes are capable of contacting and modulating a greater number of synapses and vascular units – hallmarks of their roles in maintaining CNS homeostasis and supporting the tripartite synapse.^[^ ^55^, ^56^ ^]^

The spatial distribution of these branching points was assessed by measuring the distance of each intersection from the nucleus (Figure 4g). Most intersections occurred within 20 µm of the soma, though a substantial fraction extended beyond this range. In mature astrocytes, distal branching is associated with greater coverage of synaptic fields and vascular interfaces, facilitating astrocytic involvement in extracellular ion regulation, synaptic pruning, and neuromodulatory signaling.^[^ ^57^, ^58^, ^59^ ^]^ By mapping these distances, we gain insight into the degree of astrocyte territorial expansion – an important functional indicator, especially in the context of reactive versus homeostatic phenotypes.

Together, the morphological distinctions observed across Figure 4c–g underscore the profound influence of NW topography on astrocyte development. Advanced maturation, as quantified by process number, length, and complexity, is tightly linked to the ability of these cells to fulfill their multifaceted roles in CNS physiology. While these metrics indicate that our platform recapitulates key aspects of astrocyte morphology, fine structures such as leaflets, perisynaptic astrocytic processes (PAPs), and end‐feet were not observed, highlighting differences from in vivo morphology. Overall, the measurements provide a quantitative framework for assessing astrocyte morphological states, enabling objective comparisons between experimental conditions and offering new opportunities for mechanistic investigations in neurobiology.

**Figure 5:**
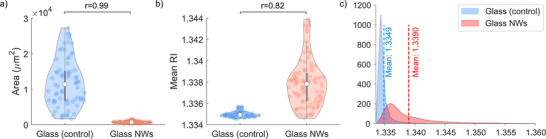
Quantitative morphological and RI analysis of astrocytes cultured on disordered glass NW substrates versus flat glass controls. Violin plots compare a) area b) mean RI of each cell with effect size (r) denoting strength of statistical significance (n = 77 cells for glass control and n = 87 cells for glass NW), c) RI distributions of astrocytes cultured on flat glass (blue) and NW substrates (red) (n = 9 cells for each type). Cells on glass display a distribution skewed toward values close to the medium (≈1.33), while astrocytes on NW exhibit a more uniform distribution shifted to higher RI values (mean ≈1.34). These differences highlight the influence of substrate nanotopography on intracellular composition.

To complement morphological characterization, we analyzed additional parameters such as RI distribution that marks the variation in localized mass density reflecting the intracellular composition and spatial organization. Astrocytes on NW substrates occupied a significantly (r = 0.99) lower projected cellular area (≈770 µm^2^) as compared to controls (**Figure** **5**a) occupying 13,800 µm^2^. The area for astrocytes on NW substrates lies within the range reported earlier (≈500–1800 µm^2^) using 3D culture.^[^ ^60^ ^]^ The more spread‐out cytoplasm for astrocytes on glass, was also accompanied with higher cytoplasmic water content indicated by both – significantly (r = 0.82) lower mean RI (≈1.335) (Figure 5b) as well as a skewed distribution with a substantial fraction of pixels having water‐like RI (≈1.33) (Figure 5c). By contrast, astrocytes on glass NWs displayed a broader, more uniform distribution shifted toward higher values (mean RI ≈1.34), consistent with reported ranges for mammalian cells^[^ ^24^, ^61^, ^62^, ^63^ ^]^ and neurons with RI of neurites being less than 1.342.^[^ ^64^ ^]^ These results indicate that substrate nanotopography can modulate not only cell morphology, but also intracellular composition reflected in RI, a proxy for local density, that underlies cellular function.^[^ ^65^ ^]^

### 2.3 Investigating Substrate Stiffness and Topography by Brillouin Microscopy

**Figure 6:**
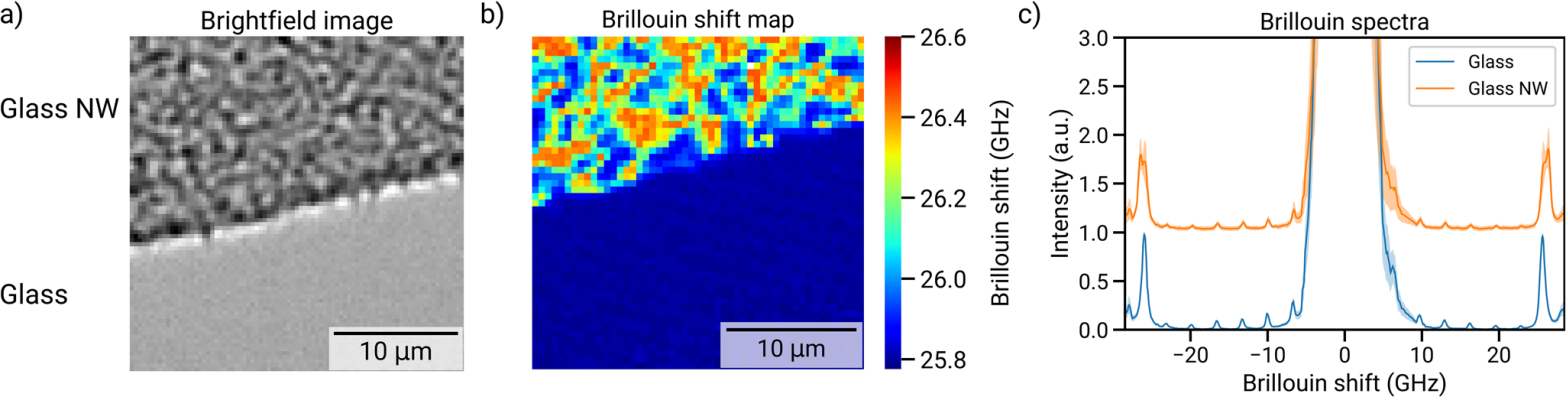
*Brillouin microscopy reveals nanoscale variations in the mechanical properties of disordered glass NW substrates*. a) Brightfield image showing regions with and without NW coverage. b) Brillouin shift map reconstructed from peak positions determined using Lorentzian fitting, highlighting spatial differences in local stiffness. c) Normalized Brillouin spectra from representative NW and bare regions; spectra are vertically offset for clarity.

To investigate whether the morphological differences observed in astrocytes were driven primarily by differences in stiffness or topographical cues, we performed Brillouin microscopy on the glass NW substrates and flat glass controls (**Figure** **6**a). Brillouin microscopy enables non‐contact, label‐free mapping of viscoelastic properties by measuring the Brillouin frequency shift, which arises from inelastic scattering of light by thermally generated acoustic phonons in the material. This frequency shift is directly proportional to the material's longitudinal modulus and serves as a sensitive proxy for local stiffness. In this study, the glass NW regions exhibited a mean Brillouin shift of ≈26.15 GHz, slightly higher than the ≈25.80 GHz measured for flat glass, indicating modestly increased stiffness in the NW regions (Figure 6b,c). Importantly, the standard deviation of the Brillouin shift in the NW areas (≈0.18 GHz) was more than ten times higher than that in the flat glass (≈0.012 GHz), revealing substantial spatial heterogeneity in mechanical properties, as also seen in the reconstructed shift map (Figure 6b).

Such heterogeneity aligns with the inherent disorder and random orientation of the NWs, and may result in a non‐uniform mechanical landscape at the cell–substrate interface. The localized mechanical variability is likely to influence focal adhesion dynamics, cytoskeletal tension, and mechanosensitive signaling in astrocytes. These findings suggest that it is not just an increase in bulk stiffness, but rather the spatial variability in mechanical cues introduced by the NWs, that drives enhanced process formation and astrocyte branching. Although a detailed mechanistic dissection of how topographical and mechanical heterogeneity cues induce in vivo‐like astrocytic morphology was beyond the scope of this study, several candidate pathways likely contribute. These include cytoskeletal remodeling processes such as actin dynamics, integrin–ECM signaling, and mechanotransductive responses to nanotopographical cues.^[^ ^66^, ^67^, ^68^, ^69^, ^70^, ^71^ ^]^ By providing a quantitative, label‐free framework to benchmark morphological outcomes, our study establishes the structural basis for future investigations that directly probe these mechanisms. Overall, the Brillouin data reinforces the conclusion that nanoscale disorder and mechanical contrast are key determinants of astrocyte morphology on engineered substrates.

## 3 Conclusion

In this study, we report the first label‐free imaging and quantitative morphological analysis of astrocytes cultured on disordered glass NW substrates using LC‐HT. This represents a key advance in the field, as no prior work has visualized astrocyte morphogenesis on such nanostructured, physiomimetic surfaces in a 3D manner using LC‐HT. Our findings show that glass NW substrates promote star‐like morphologies reminiscent of in vivo astrocytes, supporting advanced maturation and complex branching not observed on conventional flat glass. By recapitulating key mechanical and topographical features of the native ECM, these nanostructured substrates foster a microenvironment that promotes more authentic astrocyte behavior compared to conventional flat surfaces. Using LC‐HT, we captured high‐resolution, quantitative phase images of astrocytes, enabling volumetric and morphological analysis. Brillouin microscopy further revealed modest increases in stiffness and substantial spatial variation in mechanical properties across NW regions, suggesting that nanoscale disorder and mechanical contrast synergistically enhance astrocyte maturation through mechanotransductive pathways.

Overall, this work lays the foundation for a new class of nanostructured culture platforms that bridge conventional in vitro systems and the complex mechanical and topographical environments of the brain. By integrating disordered glass NW substrates with label‐free, high‐resolution imaging modalities such as LC‐HT, we present a powerful and accessible framework for studying astrocyte biology under more physiologically relevant conditions. Beyond advancing fundamental understanding of astrocyte morphogenesis and mechanosensitivity, this platform opens the door to future investigations into neurodevelopmental processes, disease modeling, and high‐content drug screening, where preserving native cell morphology and function is critical.

## 4 Experimental Section

### Fabrication of Glass NWs

SiO_2_ (glass) NWs were fabricated through thermal annealing of silicon (Si) NWs initially grown using plasma‐enhanced chemical vapor deposition (PECVD). Gold‐induced Si NWs were first grown on fused silica substrates,^[^ ^42^, ^72^, ^73^ ^]^ cut to fit the holder stage of the LC‐HT microscope. To induce the NWs growth, a 2 nm thick Au film was selectively deposited on photolithographically defined regions, followed by wet etching. Growth was conducted with SiH_4_ and H_2_ as precursors at a total pressure of 1 Torr and substrate temperature of 350 °C. The flow ratio SiH_4_/(H_2_+SiH_4_) was fixed to 1:10. Plasma was ignited using 13.6 MHz RF power at 5 W for 7 min. The resulting Si NWs formed a dense, randomly oriented mat ≈2–3 mm in length with bottom diameters of 50–80 nm. These were thermally oxidized in a controlled O_2_ atmosphere at 980 °C for 8 h to yield transparent glass NWs.^[^ ^34^, ^35^, ^36^ ^]^

### Scanning Electron Microscopy (SEM)

The morphology of the glass NWs mat was verified by a field emission scanning electron microscopy (FESEM) (ZEISS SIGMA 300) at an accelerating voltage of 5 kV. The length and diameter of the NWs were determined using line tools of the image analysis program ImageJ. Distributions were calculated from ≈500 measurements obtained from three SEM images, and histograms were generated using ≈10 bins across each distribution.

### Ultraviolet‐visible (UV/Vis) Spectroscopy

Transmittance and reflectance were measured from 300–700 nm using a Lambda 35 UV–vis spectrophotometer to evaluate optical properties.

### Atomic Force Microscopy (AFM) Characterization

AFM imaging was conducted on V1‐quality mica‐mounted glass NW substrates using a Bruker Multimode 8 AFM in tapping mode with silicon cantilevers (Bruker). The field of view was 5 µm × 5 µm, corresponding to 512 pixels × 512 pixels. The AFM map for modulus was downsampled using mean pooling with window sizes of 8 × 8 pixels and 32 × 32 pixels using the “block_reduce” function in scikit‐image^[^ ^74^ ^]^ python package, and compared to the Brillouin maps (Figure S2, Supporting Information).

### Rat Cortical Astrocyte Isolation and Purification

All animal experimental procedures were conducted in strict accordance with the Animals Care and Use Committee at Johns Hopkins University. Astrocytes were extracted from postnatal day 2 Sprague‐Dawley rats by initially isolating a neural cell mixture from the rat, followed by purification for astrocyte extraction.^[^ ^75^, ^76^ ^]^ The meningeal layer was carefully removed, and the cortical tissue was dissected in ice–cold HBSS (ThermoFisher) supplemented with 2% penicillin‐streptomycin (P/S). The wet cortex was transferred to a sterile petri dish and minced into ≈1 mm^3^ fragments using a sterile blade. These fragments were incubated in pre‐warmed 0.05% trypsin‐EDTA (ThermoFisher) at 37 °C, with gentle agitation every 5 min. After 10 min, trypsin activity was quenched with a solution containing 10% heat‐inactivated fetal bovine serum (Cytiva), 100 U mL^−1^ penicillin, 100 U mL^−1^ streptomycin (ThermoFisher), and glucose/pyruvate‐supplemented DMEM (cDMEM, ThermoFisher). The tissue was then triturated, centrifuged, and the pellet resuspended in cDMEM. This process was repeated three times, including filtration through a cell strainer to ensure a uniform cell suspension. Mixed neural cells were seeded into poly‐D‐lysine (PDL, ThermoFisher)‐coated T75 flasks (one brain per flask). PDL coating was achieved by incubating flasks with 10 µg mL^−1^ PDL (in DI water) at 0.125 mL cm^−^^2^ for 30 min at 37 °C, followed by two 5‐min DI water rinses. Cultures were initiated with 15 mL of cDMEM, with a full medium change at 24 h to remove debris. Cells were cultured for ≈1 week, with media changes every 3 days, until reaching >90% confluence. For astrocyte purification, flasks were sealed and shaken at 210 rpm for 1 h at 37 °C, followed by ≈10 firm shakes to dislodge microglia and oligodendrocytes, focusing on the flask bottom. Media was aspirated, monolayers were washed with DPBS (ThermoFisher), and non‐astrocyte removal was confirmed via microscopy. Astrocytes were then trypsinized, neutralized, and pelleted by centrifugation. The final cell suspension was washed, resuspended in serum‐free DMEM, and counted using trypan blue exclusion on a Countess automated cell counter. Astrocytes were then re‐plated onto three 1.5H poly‐D‐lysine (PDL) coated glass‐bottom petri dishes and three glass NWs substrates and cultured for 7 days, with ½ media change every 3 days. Cells were fixed on day 7, which involved 15 min of incubation at room temperature with 4% paraformaldehyde (PFA, Biotium) followed by three washes with DPBS for 5 min each. In addition, a control experiment was performed to evaluate the influence of PDL coating. Astrocytes cultured on non‐PDL coated glass were found to have similar polygonal morphology, area, and RIr distributions (Figure S1, Supporting Information).

### Astrocyte Purity Assessment

Astrocytes were characterized by immunostaining for glial fibrillary acidic protein (GFAP), a well‐established astrocytic marker. Fixed cells were permeabilized using 0.2% Triton X‐100 (Sigma–Aldrich) for 30 min at room temperature, then blocked for 1 h in blocking buffer consisting of DPBS supplemented with 5% normal goat serum (ThermoFisher), 0.2% Triton X‐100, and 1% bovine serum albumin (BSA, Sigma–Aldrich). Primary antibody incubation was performed overnight at 4 °C using anti‐GFAP (1:1000, Dako) diluted in blocking buffer. Afterward, cells were washed three times with DPBS for 10 min each and incubated with Alexa Fluor 488‐conjugated secondary antibody (1:1000, ThermoFisher) in blocking buffer for 1 h at 4 °C. Nuclei were counterstained with Hoechst 33342 (1:2000, ThermoFisher) for 10 min, followed by three 5‐min DPBS washes. Astrocyte purity was confirmed based on the majority of the cells being GFAP positive compared to the total cells with stained nucleus. Fluorescent images for GFAP and Hoest, from the same field of view, are provided in Figure S3 (Supporting Information).

### Low‐Coherence Holotomography (LC‐HT)

LC‐HT imaging was performed using the HT‐X1 system (Tomocube, Korea) with a 40x, 0.95 NA air objective lens (UPLXAPO40X, Olympus), custom‐designed condenser lens (NA = 0.72, working distance = 30 mm), and a 450 nm LED light source. Throughout the acquisition, all operations were digitally controlled and monitored by an operating software (TomoStudio X, Tomocube). At the pupil plane, a digital micromirror device is placed to apply multiple illumination profiles. At each incidence condition, a 2D hologram is recorded encoding both the intensity and phase information. The sample is also axially scanned over a range of 66 µm to collect a total of 60 stacks of the 2D holograms corresponding to the modulated illuminations. The acquired stacks are then processed to generate a 3D tomogram, through computational phase retrieval and inverse scattering algorithms based on Fourier diffraction theorem and regularization taking advantage of a priori knowledge of the specimen.^[^ ^32^, ^77^, ^78^ ^]^ The resulting 3D tomogram provided a lateral resolution of 155.4 nm and axial resolution of 0.947 µm. For HT imaging, the acquisition time is 3 ms per slice, and the illumination power is calibrated automatically, but is significantly lower than the power that would be used for fluorescence. The 2D fluorescence images were acquired with acquisition time of 80 ms (for GFAP) and 70 ms (for Hoest) with 112.5 nm resolution.

### Morphological Analysis of LC‐HT Images

The primary process of astrocytes is defined as the process that extends directly from the soma. Other processes that split from the primary processes are considered branchlets. The process lengths and soma area were calculated using the TomoAnalysis software (TomoCube, Republic of Korea). The total arbor length is defined as the sum of all the primary processes in a cell. The distance of the intersection to the nucleus was calculated by first fitting the nucleus to an ellipse and finding the center, and then measuring the distance of each intersection of processes to the center of the ellipse. The area was calculated by counting the number of non‐zero pixels in the masks. For quantitative analysis, we only included astrocytes whose soma and primary processes could be clearly distinguished; and segmented the cells manually using the 2D MIP (Maximum Intensity Projection) images in ImageJ with consistent criteria. After segmentation, n = 96 cells were obtained for glass NWs that were used for morphological analysis presented in Figure 4. For the area and mean RI analysis reported in Figure 5, n = 77 cells were used for glass (control) and n = 87 were used for glass NW after excluding cells with noisy RI voxels (due to dust in the bottom of the dishes/media). For RI distribution, representative n = 9 cells were chosen for both glass (control) astrocytes and glass NW astrocytes. To aid interpretability, correlation analysis was performed using six single‐cell features with n = 77 cells (Figure S4, Supporting Information).

### Brillouin Microscopy

Brillouin imaging was performed using a confocal Brillouin microscope equipped with a VIPA‐based hyperfine spectrometer (LightMachinery, Canada) and a tunable filter (pump killer) to suppress the main laser peak. A 660 nm single‐mode laser source (Cobolt Flamenco, Hubner Photonics) was focused using a 20x 0.8 NA objective on an inverted microscope (Nikon Eclipse Ti2). Scattered light was collected using the same objective, and recorded with a Hamamatsu Orca‐Fusion C14440 camera. 2601 spectral acquisitions were made with a 0.5 µm step size covering 25 µm x 25 µm field of view with both glass NW and glass present within the field of view. The spectra were normalized using the amplitude of the Brillouin peak and baseline corrected by offsetting the minimum value to zero.

### Statistical Analysis

Statistical significance for area and mean RI values was analyzed using Wendt formula for rank biserial coefficient, applicable with Wilcoxon rank‐sum test using MATLAB.^[^ ^79^, ^80^ ^]^ The resulting effect size values are annotated on the violin plots representing the data distribution along with the central point representing the median. The violin plots were plotted after removal of outliers using “rmoutliers” function in MATLAB.

## Conflict of Interest

The authors declare no conflict of interest.

## Supporting information



Supporting Information

## Data Availability

The data that support the findings of this study are available from the corresponding author upon reasonable request.
