# Supplementary material for: Disordered Glass Nanowire Substrates Produce in Vivo‐Like Astrocyte Morphology Revealed by Low‐Coherence Holotomography
Source: Adv Sci (Weinh). 2025 Nov 3;13(3):e13424. doi: 10.1002/advs.202513424 (PMC12806367; doi:10.1002/advs.202513424)
Supplement: Supplementary file 1 — Supporting Information [file ADVS-13-e13424-s001.docx]

Supporting Information

**Disordered glass nanowire substrates produce in vivo-like astrocyte morphology revealed by low-coherence holotomography**

Pooja Anantha^1#^, Anoushka Gupta^1#^, Joo Ho Kim^2,3^, Emanuela Saracino^4^, Piyush Raj^1^, Ivano Lucarini^5^, Swati Tanwar^1^, Jessica Chen^6^, Luo Gu^2,3,7^, Jay Agrawal^8^, Annalisa Convertino^5*^, Ishan Barman^1,9,10*^

^1^Department of Mechanical Engineering, Johns Hopkins University, Baltimore, MD 21218, USA.

^2^Department of Materials Science and Engineering, Johns Hopkins University, Baltimore, MD 21218, USA.

^3^Institute for NanoBioTechnology, Johns Hopkins University, Baltimore, MD 21218, USA.

^4^Institute for Organic Synthesis and Photoreactivity (ISOF), National Research Council, Via P. Gobetti 101, I-40129 Bologna, Italy.

^5^Institute for Microelectronics and Microsystems, National Research Council, via Fosso del Cavaliere 100, Rome, 00133 Italy.

^6^Department of Biology, Johns Hopkins University, Baltimore, MD 21218, USA.

^7^Translational Tissue Engineering Center, Johns Hopkins University School of Medicine, 400 N. Broadway, Baltimore, Maryland 21231, USA

^8^Department of Radiology, VA Hudson Valley Health Care System, Wappingers Falls, NY 12590

^9^Department of Oncology, Johns Hopkins University, Baltimore, MD 21287, USA.

^10^The Russell H. Morgan Department of Radiology and Radiological Science, Division of Cancer Imaging Research, Johns Hopkins University School of Medicine, Baltimore, MD 21205, USA.

**^#^**These authors have contributed equally.

***Co-Correspondence:**

**Annalisa Convertino**

Institute for Microelectronics and Microsystems,

National Research Council

via Fosso del Cavaliere 100

Rome, 00133 Italy

Email: annalisa.convertino@cnr.it

**Ishan Barman**,

Department of Mechanical Engineering,

Johns Hopkins University

3400 N Charles St, Baltimore, MD 21218

Office: 410-510-0656

E-mail: ibarman@jhu.edu


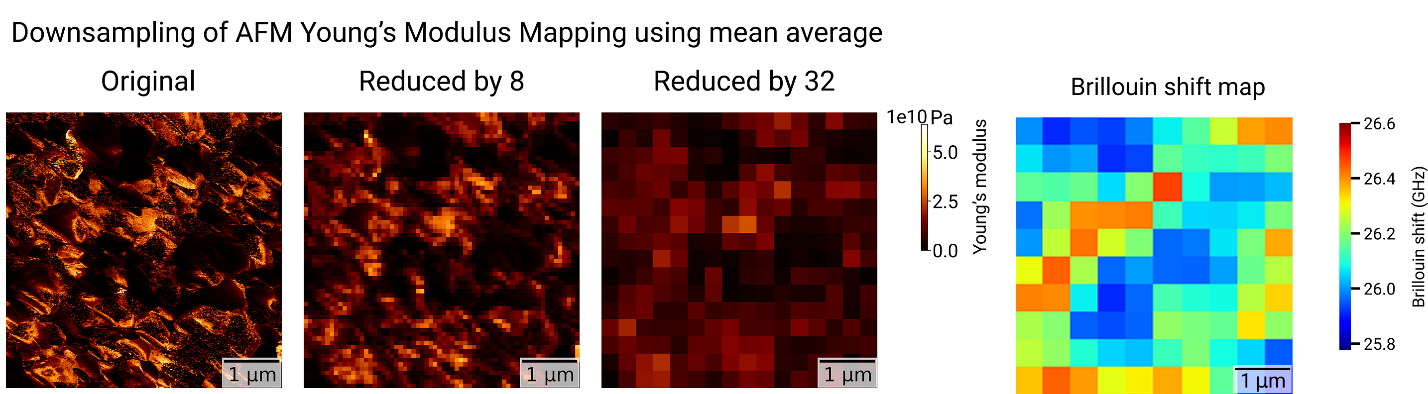


Figure S2. Original and downsampled AFM mapping of young’s modulus, along with a representative Brillouin map from a randomly chosen 5 µm x 5 µm area.


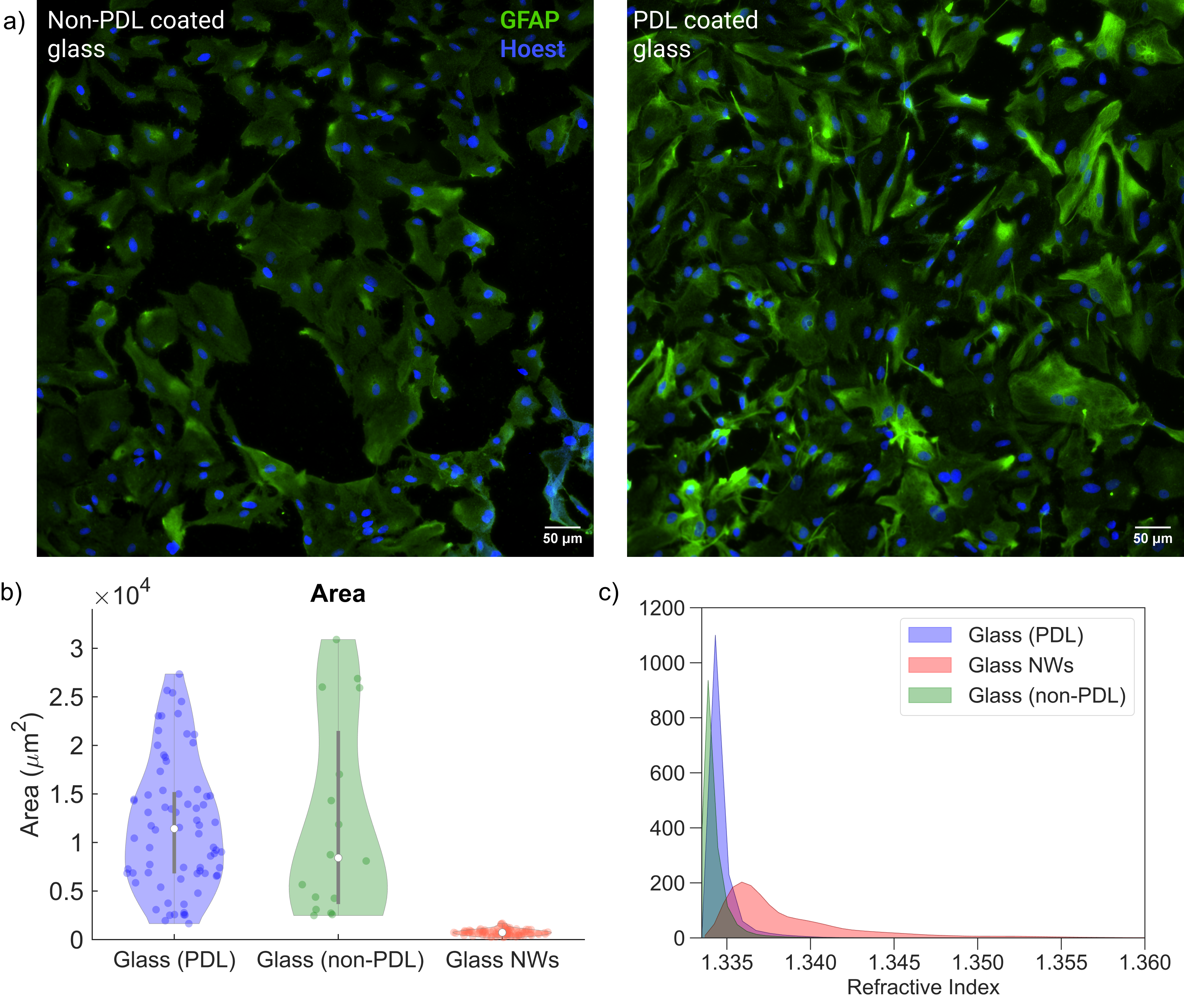


Figure S1. (a) Fluorescence images of astrocytes cultured on non-PDL coated glass (left) and. PDL-coated glass (right). (b) Both PDL-coated and non-PDL glass substrates yield comparable spreading areas (~2000 to ~30,000 µm^2^), which are substantially larger than those observed for astrocytes on glass nanowires (mean ~770 µm^2^) (n=77 cells for glass (PDL), n=16 cells for glass (non-PDL) and n=87 cells for glass NW). (c) Astrocytes on glass (PDL) and glass (non-PDL) show similar refractive index distribution indicative of swollen cytoplasm, whereas cells on glass nanowires show a distinct shift toward higher values (n= 9 cells for glass (PDL) and glass NWs, n=5 cells for glass (non-PDL)).


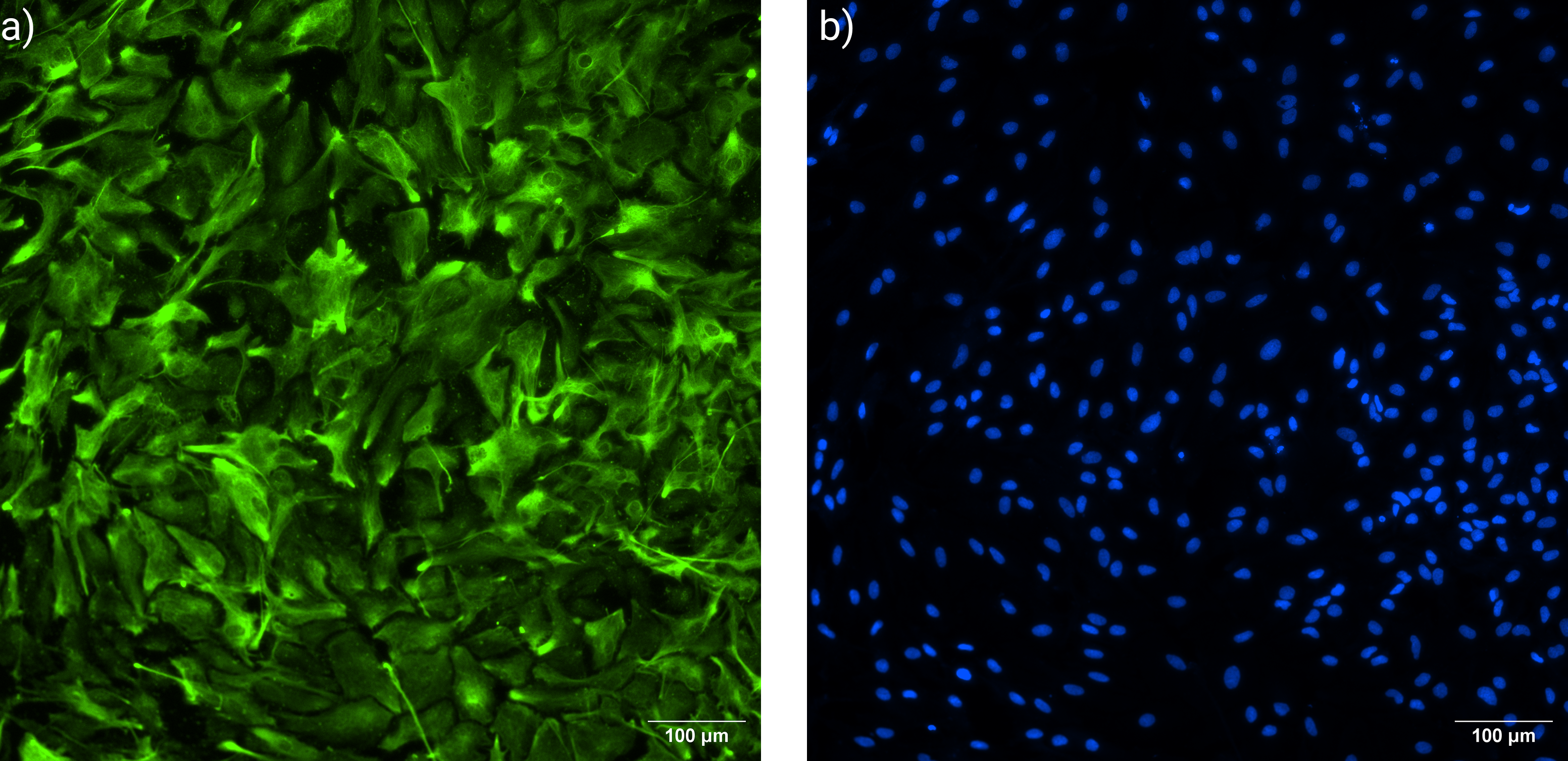


Figure S3. Representative immunofluorescence-stained images of astrocytes cultured on glass substrates to confirm culture purity. (A) Astrocytes stained for glial acidic fibrillary protein (GFAP) (B) Astrocytes stained for nucleus (Hoest). Majority of the cells present in the field of view express GFAP.


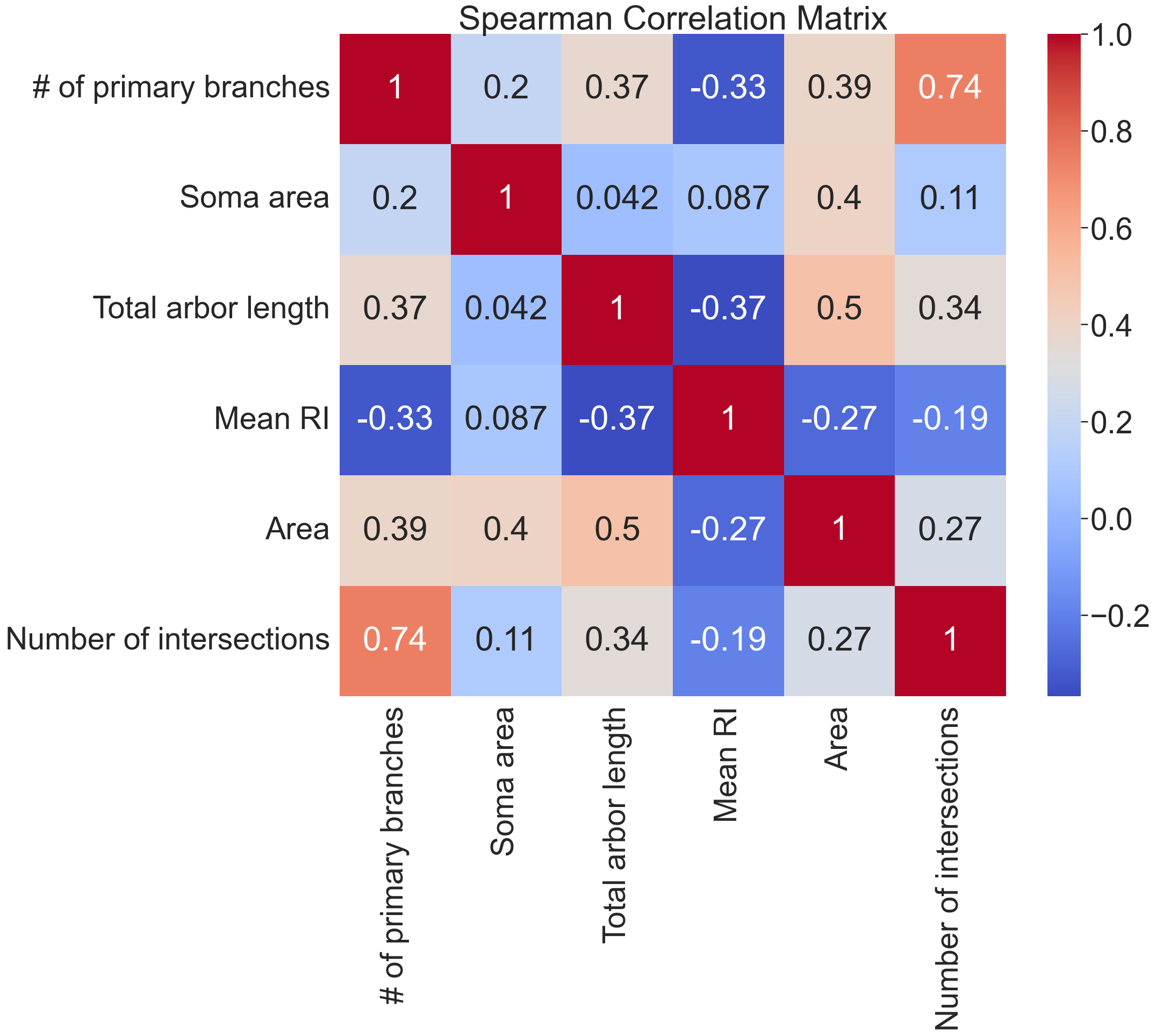


Figure S4. Correlation matrix of six features extracted from astrocytes on glass NWs (n=77 cells)
